# Supplementary material for: Learning curve and surgical outcome of robotic assisted colorectal surgery with ERAS program
Source: Sci Rep. 2022 Nov 29;12:20566. doi: 10.1038/s41598-022-24665-w (PMC9709162; doi:10.1038/s41598-022-24665-w)
Supplement: Supplementary file 2 — Supplementary Information 2. [file 41598_2022_24665_MOESM2_ESM.zip › RAL Raw data-Ñ[▒K/068/OP N_ñw╝╨░O▒Kñσ.pdf]

請選擇...reporter

臺中榮民總醫院
手術室護理記錄

[redacted]

基本資料

- 室別： 二醫手術室-31室，手術科別： CRS，手術類別： 常規，日期： 2021/06/16
- 進入時間： 0945 ； 麻醉開始時間： 0958 ； Time Out時間： 1140 ； 劃刀時間： 1141 ； 手術結束時間： 1765 送出時間： 1720 ；
- 麻醉方式： GE

  診斷：s/p LAR with dehescience.

  手術名稱：1.Low anterior resection with colo-anal anastomosis2.Ileostomy

  手術局部用藥：NIL

  術前預防性抗生素： 無
- 手術醫師： 林俊余，楊曙榮，張嘉育
- 麻醉醫師： VS 蔡志忠，麻醉醫師： R 陳昱安
-
  - 刷手護士A ： 彭馨慧，時間： 0945，刷手護士B ： 林采儀，時間： 0945，接替刷手一： 阮鈺珊/林采儀，時間： 1130/1200，接替刷手二： 朱詠暄，時間： 1500
  - 流動護士A ： 李庭 ，時間： 0945，接替流動一： 謝孟君，時間： 1145，接替流動二： 李庭，時間： 1215
- 更多接替及流動護士： 接替刷手三： 吳若綺，時間： 1600，接替流動三： 林姿岑，時間： 1500，接替流動四： 林宜旻，時間： 1700

皮膚狀況

A-V Shunt： 無

術前： 不完整

部位(術前)：

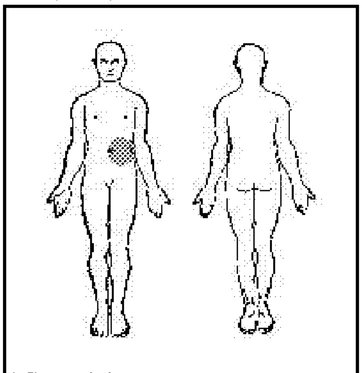

左腹1x1破皮

記錄者： 林宜旻

術後： 不完整

部位(術後)：

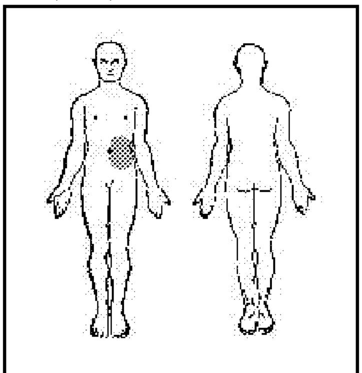

左腹1x1破皮

記錄者： 林宜旻

手術準備

手術臥式 單一手術臥式

-
    - 手術臥式： 截石臥式
    - 使用附件： 溫毯 38 ℃，頭圈，腳架
    - 皮膚準備： 10%Aq Betadine，Hibiscrub，2% Chlorhexidine
    - 電燒： 使用電燒: 電極片位置： 其它： 床上迴路電極板，記錄者： 上次簽章: 李庭

止血帶T： 無

植入物

- 植入物名稱/號碼/數量/部位： HYALOBARRIER GEL/ 30mg/ml /腸

，記錄者： 上次簽章: 林姿岑

標本送檢

- 細菌培養 厭氧，採取部位: 1. ASCITES，2. pelvis，數量： 貳
- 細胞學 無
- 冰凍切片 無
- 病理標本
  組織名稱一： Rectum
- 截肢 無

標本交付 標本： 壹 袋，標本： 零 盒，標本： 零 瓶，細胞學： 零 管，Culture Tube： 貳 管，被交付醫師： R 張嘉育，

- 記錄者： 上次簽章: 林姿岑
- 時間： 1655

傷口術後敷料

傷口敷料：

- 請於圖上標示部位：

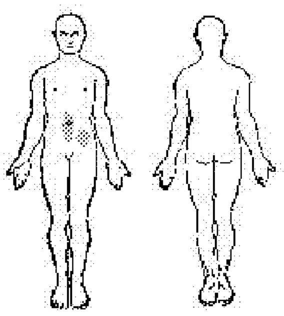

腹部直切傷口、左腹造口關閉傷口

- 傷口敷料： 白紗

傷口敷料：

- 請於圖上標示部位：

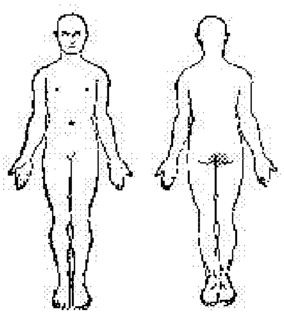

1.肛門
2.左臀Abscess 管路拔除傷口

- 傷口敷料： 1.Neomycin+散紗
  2.白紗

導管

導管	天數	導管資訊	插入日期	插入時間	更換日期	拔除日期	拔除時間	拔除原因	預計拔除/更換	預計拔除/更換日期
Sump NG Tube(1)	1	號碼:16 Fr.; 部位:胃; 註記:固定53cm;	2021/06/16	1000						
A-line(1)	1	號碼:20 G; 部位:左手;	2021/06/16	1000						
Double Lumen CVC(1)	1	號碼:8 Fr.; 部位:; 象限:RU; 註記:固定15cm;	2021/06/16	1000						
Rod,Stoma Supporting (1)	1	部位:小腸吻合處; 註記:右腹;	2021/06/16	1500						
Penrose Drain(1)	1	號碼:12 mm.; 部位:骨盆腔;	2021/06/16	1500						
Penrose										

Drain(1)	1	號碼:12 mm.; 部位:脾窩;	2021/06/16	1500						
Foley(1)	1	號碼:16 Fr.; 部位:膀胱;	2021/06/16	1120						
Chemo Port(1)	189	部位:左鎖骨;	2020/12/10	1000						

用物計數

- 手術中出血量： 200 c.c.
- 手術中尿量： 500 c.c.
-
    - 其它： 無

用物計數：

- 部位一： 肛門，1.器械： 正確，2.縫針： 正確，3.刀片： 未使用，4.紗布： 正確，5.腹部墊： 未使用，6.紗球： 未使用，7.PEANUTS： 未使用，8.紗條： 未使用，9.絲帶： 未使用，10.針頭： 未使用，11.Vessel loop： 未使用，12.Boots： 未使用，13.Jelco： 未使用，14.棉條： 未使用，
    - 第一次：
        - 刷手護士 ： 阮鈺珊/林采儀
        - 流動護士： 上次簽章: 李庭
        - 時間： 1440

  ,
    - 第二次：
        - 刷手護士 ： 阮鈺珊/林采儀
        - 流動護士： 上次簽章: 李庭
        - 時間： 1450

  ,
    - 第三次：
        - 刷手護士 ： 阮鈺珊/林采儀
        - 流動護士： 上次簽章: 李庭
        - 時間： 1500
- 部位二： 腹部，1.器械： 正確，2.縫針： 正確，3.刀片： 正確，4.紗布： 正確，5.腹部墊： 正確，6.紗球： 未使用，7.PEANUTS： 未使用，8.紗條： 未使用，9.絲帶： 正確，10.針頭： 正確，11.Vessel loop： 未使用，12.Boots： 未使用，13.Jelco： 未使用，14.棉條： 未使用，
    - 第一次：
        - 刷手護士 ： 朱詠暄
        - 流動護士： 上次簽章: 林姿岑
        - 時間： 1515

  ,
    - 第二次：
        - 刷手護士 ： 朱詠暄
        - 流動護士： 上次簽章: 林姿岑
        - 時間： 1530

  ,
    - 第三次：
        - 刷手護士 ： 吳若綺
        - 流動護士： 上次簽章: 林姿岑
        - 時間： 1603
-
    - 第一次：
        - 流動護士：

  ,
    - 第二次：
        - 流動護士：

  ,
    - 第三次：
        - 流動護士：

備註

備註：NIL

- 記錄者： 林宜旻 上次簽章: 林姿岑
- 時間： 1715

填寫護理人員 林宜旻

正式摘要

		請選擇...reporter			

doctorRank:B
